# Percutaneous endoscopic interlaminar decompression for degenerative scoliosis in the elderly: a safe and effective minimally invasive alternative

**DOI:** 10.3389/fsurg.2025.1667811

**Published:** 2025-10-21

**Authors:** Bin Zheng, Panfeng Yu, Tiepeng Han, Haiying Liu, Yan Liang

**Affiliations:** Spine Surgery, Peking University People’s Hospital, Beijing, China

**Keywords:** degenerative scoliosis, elderly patients, percutaneous endoscopic interlaminar decompression, minimally invasive surgery, spinal stenosis

## Abstract

**Background:**

Elderly patients with degenerative scoliosis combined with spinal stenosis present significant treatment challenges. Traditional open fusion surgery carries high risks and complications in this population. This study investigates the clinical efficacy and safety of Percutaneous Endoscopic Interlaminar Decompression (PEID) as a potential minimally invasive alternative.

**Methods:**

A retrospective case series of 32 elderly patients (≥60 years) with degenerative lumbar scoliosis (Cobb angle 10°–30°) undergo PEID between January 2022 and December 2023. All procedures are performed under general anesthesia without internal fixation. Clinical outcomes are assessed using Visual Analog Scale (VAS) for pain and Oswestry Disability Index (ODI) for functional status. Radiographic evaluation includes measurement of scoliosis Cobb angles and adjacent intervertebral height. Follow-up is conducted at 1 week, 1 month, and 1 year postoperatively.

**Results:**

The study includes 14 males and 18 females with a mean age of 68.5 ± 6.1 years. The average operative time is 99.26 ± 13.17 min. VAS scores improved significantly from 7.4 ± 1.3 to 2.1 ± 0.7, and ODI from 55.0% ± 10.2% to 15.8% ± 5.0% at the final follow-up (both *P* < 0.01).According to modified Macnab criteria, 93% of patients achieve excellent or good outcomes. Minor complications occur in 2 patients (6.25%) with no major neurological complications. Radiographic analysis shows no significant progression of scoliosis (Cobb angle: 15.4° ± 6.2° vs. 15.7° ± 6.5°, *P* > 0.05) and no evidence of adjacent segment degeneration.

**Conclusions:**

PEID provides effective symptom relief and functional improvement for elderly patients with degenerative scoliosis, while maintaining spine stability. This minimally invasive approach offers a safe alternative to traditional open surgery in carefully selected patients with moderate scoliosis and primarily compressive symptoms.

## 1 Introduction

Degenerative scoliosis is typically defined as spine curvature that appears after adult skeletal maturity, with a coronal Cobb angle >10° on x-ray, accompanied by degenerative changes in intervertebral discs and facet joints. DLS is commonly seen in elderly patients and often coexists with lumbar spinal stenosis (1, 2). Scoliotic deformity and spinal stenosis caused by spine degeneration can compress neural structures, causing clinical symptoms such as low back pain, lower limb radicular pain, and intermittent claudication, severely affecting patients’ quality of life (3).

For elderly patients with degenerative spine scoliosis who have obvious symptoms, conservative treatment (such as physical therapy, analgesics, nerve blocks, etc.) often fails to achieve satisfactory results. Traditional open surgery often requires extensive spine decompression and internal fixation fusion to simultaneously address neural compression and spine stability issues (4). However, in elderly patients, long-segment fixation fusion surgery is highly traumatic with high complication rates, and even carries high risks of perioperative complications and mortality (5). Additionally, due to factors such as spine stiffness and osteoporosis in elderly patients, even fusion and corrective surgery may be difficult to achieve ideal correction results, and postoperative complications such as internal fixation loosening, correction loss, or pseudarthrosis formation may occur (6). Furthermore, spine fusion surgery faces long-term risks of adjacent segment degeneration (ASD), with studies showing that the incidence of adjacent segment radiographic degeneration can reach 33.6% within 2–5 years after fusion surgery (7). Therefore, for moderate degenerative scoliosis with Cobb angles of 10°–30°, some scholars suggest that extensive long-segment fusion fixation surgery is unnecessary (8, 9). Finding a treatment method that can adequately decompress and relieve symptoms while minimizing surgical trauma and complications is of great significance for these elderly patients.

In recent years, advances in minimally invasive spine surgery techniques have provided new approaches for treating elderly degenerative spine scoliosis. Among these, percutaneous PERCUTANEOUS ENDOSCOPIC INTERLAMINAR DECOMPRESSION can complete spine decompression under general anesthesia. PEID avoids large incisions and extensive muscle dissection of the posterior midline approach, maximally preserving the integrity of spine bony structures with minimal impact on spine stability (10). Compared with traditional open surgery, PEID has advantages including small surgical incisions, less bleeding, mild postoperative pain, and rapid recovery (11).

This study observes the clinical efficacy and safety of PEID applied to elderly patients with degenerative spine scoliosis combined with spinal stenosis, and summarizes the value of minimally invasive surgery in this type of disease.

## 2 Materials and methods

### 2.1 Study subjects

This study is a retrospective case series analysis. We collect 32 elderly patients with degenerative scoliosis combined with spinal stenosis who are admitted to our hospital from January 2022 to December 2023 and meet the inclusion criteria. All surgeries are performed by a spine surgeon with more than 10 years of experience in endoscopic surgery.

Inclusion criteria: ① Age ≥60 years; ② Imaging confirmation of lumbar degenerative scoliosis (Cobb angle >10°) with spinal stenosis or nerve root canal stenosis, consistent with clinical symptoms; ③ No significant improvement after at least 3 months of standard conservative treatment, requiring surgical decompression; ④ Coronal Cobb angle <30° with no severe sagittal imbalance; ⑤ No obvious spine instability preoperatively. ⑥ Single-level surgery.

Exclusion criteria: severe osteoporosis (bone density *T*-score < −2.5 with high fracture risk), combined spine infection or tumor, previous open surgery history at the same segment, systemic medical diseases unable to tolerate surgery, etc.

Among the 32 included patients, there are 14 males and 18 females, aged 60–82 years, with an average age of (68.5 ± 6.1) years. All patients mainly present with varying degrees of low back pain, with or without unilateral/bilateral lower limb pain and numbness, and intermittent claudication. Preoperative imaging examination confirms that all patients have lumbar degenerative scoliosis [average coronal Cobb angle (15.4 ± 6.2)°] with signs of spinal stenosis or nerve root compression. All patients undergo surgery after conservative treatment (including medication, physiotherapy, etc.) proves ineffective. Surgery is performed by the same spine surgery team, and informed consent is obtained from all patients preoperatively.

### 2.2 Surgical method

The responsible spinal segment was identified preoperatively based on clinical symptoms, physical examination, and imaging findings. When diagnosis was uncertain, a selective nerve root block was performed as confirmation.

All patients underwent posterior percutaneous endoscopic interlaminar decompression (PEID) under general anesthesia in the prone position. The choice of the interlaminar approach was based on the anatomical characteristics of elderly degenerative scoliosis patients: most presented with foraminal stenosis and a relatively preserved interlaminar window, making the interlaminar route safe and more direct than the transforaminal approach. In addition, the symptomatic side was preferentially selected as the entry side, and the approach was adjusted according to the width of the interlaminar space and the location of stenosis.

Under C-arm fluoroscopic guidance, a working cannula was placed into the interlaminar window of the target segment. The endoscope was then inserted through the cannula into the spinal canal. Under direct visualization, hypertrophied ligamentum flavum, bony overgrowth at the lamina or facet joint, and herniated disc material were sequentially removed to decompress the lateral recess and nerve root canal. For patients with central stenosis or bilateral symptoms, bilateral decompression through a unilateral interlaminar approach was performed when feasible. For patients with central stenosis or bilateral neurological symptoms, a bilateral decompression through a unilateral interlaminar approach is performed using the standard “over-the-top” technique. After completing ipsilateral decompression, the endoscope and instruments are angled medially across the midline to resect the contralateral hypertrophied ligamentum flavum and decompress the contralateral lateral recess and canal. Throughout the procedure, the dural sac and nerve roots were carefully protected to avoid traction or thermal injury.

### 2.3 Follow-up and assessment

Postoperatively, all patients receive routine antibiotic prophylaxis for 1 day and symptomatic analgesia. Patients are encouraged to ambulate on postoperative day 1 with lumbar support. Discharge criteria include independent walking without significant worsening of lower limb neurological symptoms. All patients are followed up for 1 year, including clinical symptom improvement and imaging examination. Follow-up visits are scheduled at 1 week, 1 month, and 1 year postoperatively. Clinical efficacy assessment indicators include low back and leg pain Visual Analog Scale (VAS, 0–10 points) and Oswestry Disability Index (ODI). VAS scores and ODI indices are recorded preoperatively and at each follow-up visit and compared with preoperative values. At the final follow-up, overall clinical efficacy is evaluated according to modified Macnab criteria, with results classified as excellent, good, fair, or poor. Imaging assessment includes standing full-spine x-rays preoperatively and at follow-up, measuring Cobb angle and adjacent intervertebral height. Adjacent intervertebral height represents the degeneration of adjacent segments.

### 2.4 Statistical analysis

SPSS 26.0 statistical software is used for data analysis. Quantitative data are expressed as mean ± standard deviation. VAS and ODI values at different time points before and after surgery are compared using repeated measures analysis of variance, with *post-hoc* LSD method for pairwise comparisons. *P* < 0.05 is considered statistically significant.

## 3 Results

### 3.1 General conditions and surgery-related indicators

A total of 32 patients are included in this group, all of whom successfully complete PEID. There are 14 males and 18 females with an average age of (66.68 ± 4.36) years and BMI of (23.2 ± 1.88) kg/m^2^. All surgeries are completed under general anesthesia with an average operative time of (99.26 ± 13.17) minutes. No serious complications such as nerve root injury or dural tear occur postoperatively. There are 2 cases of mild perioperative complications: 1 case of worsened ipsilateral calf numbness, which resolves after 2 weeks of neurotrophic and steroid treatment; 1 case of incision infection, which heals after dressing changes and antibiotic treatment. No patients require reoperation.

### 3.2 Pain and functional scores

**Figure 1:**
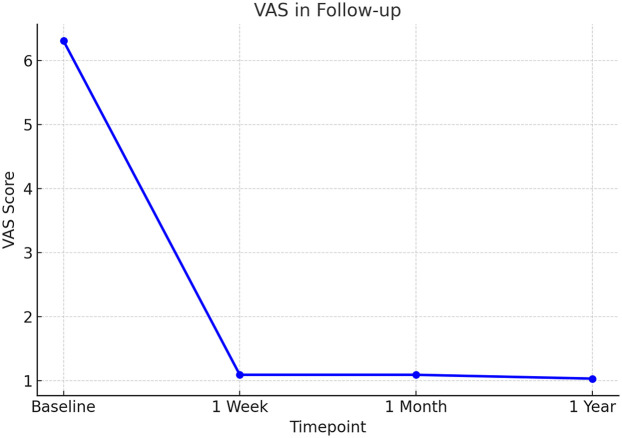
Visual Analog Scale (VAS) scores during follow-up after PEID. VAS scores for low back and leg pain decreased significantly from baseline to postoperative follow-up. A sharp reduction was observed at 1 week, and symptom relief was maintained at 1 month and 1 year.

**Figure 2:**
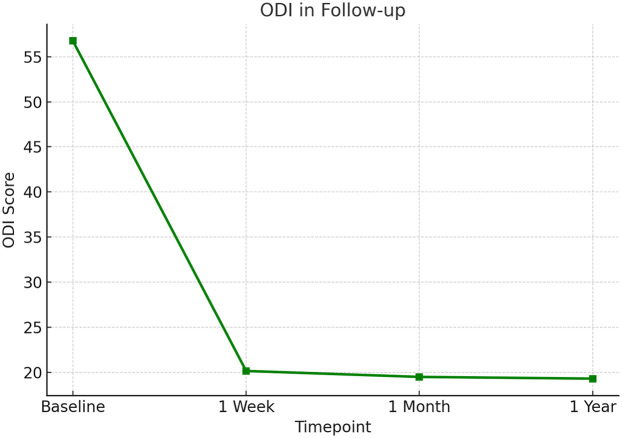
Oswestry Disability Index (ODI) scores during follow-up after PEID. ODI scores improved markedly following surgery, showing a steep decline at 1 week and remaining stable at low levels at 1 month and 1 year, indicating sustained functional recovery.

The clinical follow-up results are summarized in Table 1. Preoperative low back and leg pain VAS score is (7.4 ± 1.3), which decreases to (3.1 ± 0.9) at 1 week postoperatively, (2.4 ± 0.8) at 1 month postoperatively, and (2.1 ± 0.7) at final follow-up; all follow-up time points show significant improvement compared to preoperative values (all *P* < 0.01). ODI decreases from preoperative (55.0 ± 10.2)% to (30.5 ± 8.6)% at 1 week postoperatively, (20.4 ± 6.5)% at 1 month postoperatively, and (15.8 ± 5.0)% at final follow-up (all *P* < 0.01). Scores at 1 week and 1 month postoperatively show significant improvement compared to preoperative values, and there is no statistical difference between final follow-up and 1 month postoperatively (*P* > 0.05), indicating that efficacy can be maintained. The trend of ODI and VAS improvement during follow-up is shown in Figures 1, 2, respectively. Both curves demonstrate a sharp decline after surgery, with stable maintenance of symptom relief and functional improvement at 1-year follow-up.

**Table 1:** Clinical outcomes in follow-up.

Variable	VAS	*P* value	ODI	*P* value
Baseline	6.31 ± 1.12	*P* < 0.001	56.75 ± 4.60	*P* < 0.001
1 week postoperative	1.09 ± 1		20.16 ± 3.08	
1 month postoperative	1.09 ± 0.86		19.5 ± 3.58	
1 year postoperative	1.03 ± 1.03		19.31 ± 2.99	

### 3.3 Overall clinical efficacy assessment

Clinical efficacy is assessed according to modified Macnab criteria at final follow-up: excellent in 22 cases (68%), good in 8 cases (25%), fair in 2 cases (7%), and poor in 0 cases; overall excellent and good rate is 93.0%. Most patients are satisfied with postoperative symptom improvement and functional recovery.

### 3.4 Radiographic assessment

The radiographic outcomes follow-up are followed in Table 2. Preoperative lumbar scoliosis Cobb angle is (15.4 ± 6.2)°, and (15.7 ± 6.5)° at final follow-up, with no statistically significant difference between the two (*P* > 0.05), indicating no obvious progression of scoliosis during follow-up. Adjacent segment disc height also shows no obvious decrease or loss, suggesting no obvious new degeneration in adjacent segments when only PEID is performed without internal fixation.

### 3.5 Typical case

**Figure 3:**
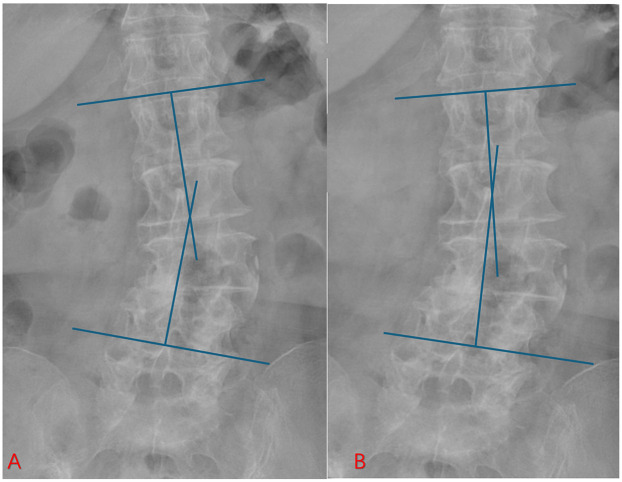
Preoperative and postoperative radiographs of an elderly patient with degenerative scoliosis. **(A)** Preoperative lumbar x-ray showing coronal deformity with degenerative changes. **(B)** One-year postoperative follow-up radiograph after PEID. The coronal Cobb angle remained stable without progression of scoliosis, indicating that the PEID effectively relieved symptoms without accelerating deformity progression.

**Figure 4:**
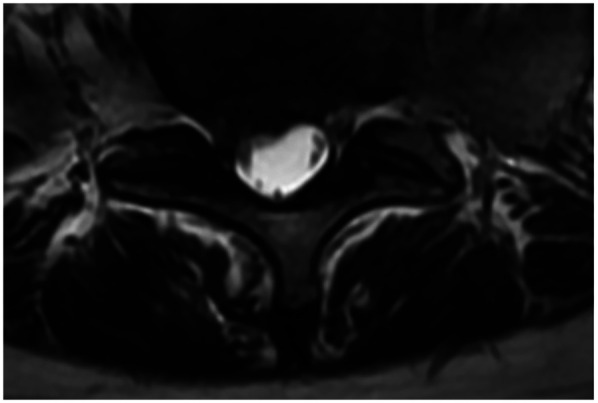
Preoperative and postoperative axial T2-weighted MRI scans. **(A)** Preoperative image showing a herniated intervertebral disc (red arrow) compressing the nerve root. **(B)** Postoperative follow-up demonstrating adequate decompression, with the nerve root clearly released and no residual compression at the surgical level.

This classic case involves a 71-year-old woman with degenerative lumbar scoliosis (coronal Cobb angle 18°, Figure 3A) who presented with low-back pain and right-leg numbness caused by right-sided L4–L5 lateral recess stenosis. Under general anesthesia she underwent precise decompression via a right interlaminar PEID approach, with an operative time of only 90 min and ∼30 ml blood loss, and no neural or dural complications; she was able to ambulate 6 h post-operatively. Pre-operative MRI (Figure 4A) vs. post-operative MRI (Figure 4B) shows adequate decompression. At 1-year follow-up her leg-and-back VAS had fallen to 1 point and ODI to 20%, while the Cobb angle remained 17° (Figure 3B).

**Table 2:** Radiographic outcomes in follow-up.

Variable	Coronal Cobb	*P* value	Adjacent segment height	*P* value
Baseline	20.77 ± 3.21	*P* = 0.88	12.29 ± 1.4	*P* = 0.6
1 week postoperative	20.83 ± 3.25		12.25 ± 1.6	
1 month postoperative	20.90 ± 3.44		12.27 ± 1.91	
1 year postoperative	20.91 ± 3.68		12.32 ± 2.21	

## 4 Discussion

Degenerative scoliosis often coexists with spinal stenosis, and the treatment goal is to relieve neural compression symptoms while maintaining spine alignment and balance. Treatment selection for such patients requires comprehensive consideration of age, degree of curvature, symptom severity, and general condition. Previously, for cases with severe symptoms, posterior decompression and long-segment internal fixation corrective fusion is often preferred to simultaneously relieve neural compression, correct deformity, and reconstruct stability (12). However, major fusion surgery carries high risks in elderly patients, with domestic and international studies reporting complication rates for elderly spine scoliosis surgery far higher than the general population, even reaching 40%-80% (13). Moreover, fusion surgery may accelerate adjacent segment degeneration, leading to long-term problems (14). In this study, we use PEID decompression without fusion fixation to treat elderly degenerative scoliosis combined with spinal stenosis, achieving good clinical results. Results show that patients' postoperative pain and function are significantly improved and maintained at 1-year follow-up, while lumbar scoliosis Cobb angle shows no significant change during follow-up, and spine stability and adjacent segment conditions are good (15, 16). This suggests that for degenerative spine scoliosis patients with small Cobb angles, PEID is a feasible treatment option that can achieve adequate decompression while avoiding risks associated with major surgery.

The success of PEID in elderly patients with degenerative lumbar scoliosis largely depends on careful patient selection. Beyond the basic inclusion criteria, several clinical and radiological factors guide the decision-making process.

First, the definition of “no obvious spinal instability” requires clarification. In this study, instability is excluded when there is no dynamic translation >3 mm or angular motion >10° on flexion–extension radiographs. In addition, the absence of significant sagittal imbalance, such as a sagittal vertical axis exceeding 5 cm, is considered an essential prerequisite. Facet joint preservation on CT and the lack of high-grade spondylolisthesis also support stability.

Second, it is crucial to distinguish symptoms caused primarily by neural compression from those resulting from deformity. Patients who present with predominant radicular pain, neurogenic claudication, or imaging-confirmed stenosis that correlates with symptoms are considered better candidates than those whose main complaint is cosmetic deformity or coronal imbalance. Diagnostic selective nerve root blocks play an important role in this differentiation. Relief of leg pain after injection confirms the responsible level and ensures that decompression targets the correct symptomatic segment.

The reason PEID can achieve satisfactory results in elderly degenerative spine scoliosis is mainly due to its minimally invasive and precise decompression characteristics. On one hand, PEID avoids large incisions and extensive posterior muscle dissection. It preserves spinal bony structures to the greatest extent while minimizing the impact on spinal stability. Patients in this group generally recover quickly, are able to ambulate within 1 day, and have an average postoperative hospital stay of less than 5 days, confirming the accelerated recovery advantages of minimally invasive surgery. On the other hand, endoscopic magnified vision and fine instruments make decompression operations safe and efficient, allowing thorough removal of compressive factors under direct visualization while protecting normal bone and joint structures, thereby avoiding iatrogenic instability. Particularly when treating scoliosis patients, endoscopic technology can select convex or concave side approaches for targeted decompression of affected segments, unlike open surgery which requires extensive dissection and wide exposure, causing less interference with overall spine balance. It is worth noting that none of the cases in this study undergo simultaneous fusion fixation, but no deterioration in spine stability or worsening scoliosis is observed during 1-year follow-up, suggesting that for appropriately selected cases, simple decompression does not significantly promote deformity progression. This is consistent with views reported in some literature that for degenerative scoliosis patients with Cobb angles ≤30°, decompression surgery can achieve good results without simultaneous fusion correction.

This study has several limitations. First, it is a retrospective case series without a control group, which reduces the strength of causal inference. Second, all included patients had moderate scoliosis angles and no severe instability, so the findings may not be generalizable to patients with larger deformities or marked sagittal imbalance. Third, the follow-up duration was limited to 1 year, preventing assessment of longer-term outcomes such as late progression of scoliosis or adjacent segment degeneration. In addition, PEID has a certain learning curve, and all procedures in this study were performed by a single experienced endoscopic surgeon, which may limit the generalizability of results. In addition, sagittal parameters such as lumbar lordosis and PI–LL mismatch are not assessed, which limits the comprehensiveness of radiographic evaluation. Future studies should include sagittal alignment to provide a more complete understanding of postoperative balance.

Future studies should include sagittal alignment parameters, adopt prospective controlled designs with larger sample sizes, and extend follow-up to better evaluate long-term outcomes such as scoliosis progression, sagittal balance, and adjacent segment degeneration. Comparative studies with other minimally invasive or fusion techniques are also needed to clarify the relative advantages and indications of PEID.

## 5 Conclusion

PEID for treating elderly degenerative lumbar scoliosis combined with spinal stenosis can achieve good clinical results and safety. This technique can significantly relieve patients' low back and leg pain symptoms, improve daily function, and postoperative follow-up observation shows no further progression of spine scoliosis, with good stability of operated segments and no obvious degeneration in adjacent segments. For elderly patients with moderate spine scoliosis primarily presenting with neural compression symptoms, PEID is a beneficial treatment option.

## Data Availability

The raw data supporting the conclusions of this article will be made available by the authors, without undue reservation.
